# Shaping the Post-COVID-19 Agenda: A Call for Responsible Leadership: How the COVID-19 Crisis Could Accelerate the Transition to a New Era for Society When Its Leaders Take Responsibility to Establish the New Common

**DOI:** 10.1007/978-3-030-65355-2_15

**Published:** 2021-03-20

**Authors:** Ronald de Jong, Mirjam van Reisen

**Affiliations:** 1 Tilburg University, Tilburg, The Netherlands; 2 Tilburg University, Tilburg, The Netherlands; 3 Tilburg University, Tilburg, The Netherlands; 4 Tilburg University, Tilburg, The Netherlands; 5 Department of Marketing, Tilburg School of Economics and Management, Tilburg, The Netherlands; 6 Department of Culture Studies, Tilburg School of Humanities and Digital Sciences, Tilburg, The Netherlands

## Abstract

In 1970, the late economist and Nobel Prize Winner Milton Friedman spread a doctrine that has dominated the business world ever since: that a company’s solitary purpose is to increase financial value for its shareholders. Author during the height of the Cold War, Friedman tied in with the narrative that economic freedom is essential to political freedom. Friedman died in 2006, but his mantra did not. From the beginning of the 1990s until the financial crisis in 2007, the business world increasingly witnessed a movement towards the “Anglo-American” or “neo liberal” model, based upon Friedman’s doctrine. In the period after the collapse of Lehmann Brothers, leaders in the public and the private sector tried to fight the financials crisis by holding on to the very same measures and instruments that—arguably—caused the financial crisis in the first place. The last convulsions of Friedman’s doctrine?

In 1970, the late economist and Nobel Prize Winner Milton Friedman spread a doctrine that has dominated the business world ever since: that a company’s solitary purpose is to increase financial value for its shareholders. Author during the height of the Cold War, Friedman tied in with the narrative that economic freedom is essential to political freedom.

Friedman died in 2006, but his mantra did not. From the beginning of the 1990s until the financial crisis in 2007, the business world increasingly witnessed a movement towards the “Anglo-American” or “neo liberal” model, based upon Friedman’s doctrine. In the period after the collapse of Lehmann Brothers, leaders in the public and the private sector tried to fight the financials crisis by holding on to the very same measures and instruments that—arguably—caused the financial crisis in the first place. The last convulsions of Friedman’s doctrine?

## The Need to Challenge Capitalism in Its Current Form

There are many symptoms indicating the end of the industrial age as we know it. We seem to begin to realize that shareholders’ interests are not necessarily and not always aligned with the interests of other stakeholders and of society at large. Value creation at the expense of instead of in harmony with is increasingly seen as unacceptable and wrong. It is increasingly inescapable to conclude that applying the paradigms of the last century to welfare creation will lead to big and potentially irreversible problems such as social unrest, increasing economic inequality, extreme poverty, and climate change destruction. This, in turn, undermines the long-term relevance of “capitalism” as the dominant economic doctrine.

## The COVID-19 Crisis as Trigger

The COVID-19 crisis is a focusing event. We have suddenly landed in a new universe. Unexpectedly and in a brief moment, what is essential to our societies has been redefined. The author John Kingdon (1984) saw such moments in time as windows when new issues could enter the policy agenda. We have come to realize that vital workers (e.g., health workers, teachers, and the police) are among those that had been subject to cuts in the public sector for decades; that health and education are vital public institutions that might not be best managed by the market; that the collective is more important than the individual; and that we are all dependent upon each other and the world around us, so we all need to be well in order to be well on an individual level.

A moment in time when all existing “certainties” are up in the air should lead to a disruption of existing doctrines and paradigms. The COVID-19 crisis, with the images of sickness and death, suddenly reveals that life, dignity, and care for family and communities are values of a higher order than the value of financial gains alone. The crisis forces business leaders to rethink the purpose of their organizations. This intensifies the debate around the role of corporate enterprise vis à vis its stakeholders. Shareholder capitalism is deeply at odds with what has become essential in the COVID-19 crisis. There is no alternative but for capitalism to reinvent its underlying paradigms and parameters and to become inclusive.

## Towards Inclusive Capitalism

Inclusive capitalism is based upon the conviction that conventional economic thinking will have to be disrupted rather than finding a justification for the enormous and increasing inequality. Value creation will have to be redefined and realized along three core dimensions: social, environmental, and economic sustainability. Business leaders will need to lead the transition to a new reality where we share everything more fairly and equally and where we live within the means of what this planet and its people have to offer. The extractive and exploitative system must make way for a new economic logic where we responsibly and purposefully lead our organizations to serve all stakeholders, where we aim to create ecological and social value alongside economic value, and where we share the latter more fairly and equally. A system change is needed, geared to protecting the commons: the common resources that we share as a society. We need to actively protect these.

This idealism is realism. Perhaps we need to rethink fossil fuel with a negative price on the market since we all know that we need to end the fossil fuel economy. Perhaps we need to rethink the provision of subsidies by the International Monetary Fund to the fossil fuel industries of 5000 million USD a year (Coady et al. 2019). Perhaps we need to rethink the state support provided to airlines because these are some of the bigger contributors to CO_2_ pollution. And perhaps we need to rethink the tax derogation to big internet platform companies, so that we can support wealth redistribution. Companies need to become part of the solution. It is time for the public and private sectors to join forces and unite around the common purpose of assuming responsibility. The Paris Climate Treaty helps set the objectives. This includes taking accountability for assisting vulnerable people and countries and to provide sustainable solutions for the current problems that are mere symptoms of much bigger—underlying—issues.

The business world needs to orient itself to addressing the big wicked problems society is now facing by aligning their missions, visions, ambitions, and strategies to contribute to their solutions. The execution will require innovative business models. Business and performance management systems, tools, and methodologies will have to be reinvented to support a new way of defining, measuring, and managing business performance based on the three core dimensions. The Sustainable Development Goals of the United Nations provide a compass for this. Instead of exclusion and polarization, Europe should focus on working with others beyond its borders, particularly with Africa, to strengthen a joint mission of international responsibility to eradicate poverty, protect the earth, and create inclusive wealth.

## Joining Forces to Create the New Common

A new generation of responsible leaders is already redefining the role of a corporation in society. In 2019, the most powerful US corporate lobby, the Business Roundtable, jettisoned the “Friedman model.” The chief executives of 181 public companies pledged to care for the environment and create value for all their stakeholders (customers, employees, suppliers, and society) as well as distribute the created value more equally (to be fair is to share).

We see the societal impact of COVID-19 unfolding as demonstrations for social justice grip our world. The depth of the crisis could be either an obstacle or an accelerator. This crisis is therefore a litmus test to proof that the intent of corporations to creating shared value is real. There are concerns that putting purpose before profit will fall by the wayside when economies are on the brink. However, a return to shareholder capitalism, as we know it, will be a colossal mistake and a missed opportunity to accelerate the transition to a new era, which we so desperately need.

No one can do this alone. We need governments, multi-lateral organizations, universities, non-governmental organizations, multinational corporations, and small and medium-sized enterprises to join forces. Amongst all of this, the COVID-19 crisis shows that we might need governments to take a more proactive and prominent role in changing the rules that help businesses to contribute to shared value for our societies. Three points are critical to achieving this:

1. A reorientation of the role of the market as a regulator for the common good and general purpose. Inclusive capitalism needs to ensure we correct the flaws of shareholder capitalism and critically review the role of governments as regulators of critical public sectors, such as health care, energy, education, and public transportation.
2. A transition to a strong European common, in which individual countries overcome their differences and join forces as we enter this new era. European countries have a special obligation and duty in this context and “Team Europe” has a unique obligation to play a leading role in shaping the new common and filling the void, as this is the first time in modern history that the United States is not assuming a leading role on a global scale. Traditional European values, in which businesses, governments, and citizens are stakeholders seeking to balance interests (commonly known as the Rhineland model) is much needed in this transition.
3. A conditionality of economic state support given to enterprises that ran into trouble due to COVID-19 to prevent supporting the status quo or return to the “old normal” but instead using such support to accelerate the transition to a new common guided by the Sustainable Development Goals and the Paris Climate Treaty.

## A Call for Responsible Leadership

With respect to corporations, there is a growing sense that profit and purpose can no longer exist in isolation: alignment will ensure success both now and in the future. Creating shared value is a moral imperative, but it has a strong business case too. An indication is the resilience of ethical investment funds with more than half of them outperforming their benchmark in March in the earlier stages of the coronavirus pandemic and attracting inflows despite the uncertainties.

Responsible leaders are accountable for more than just the short-term individual financial results of their organizations or their short-term gains. They understand their obligation to help society protect the vulnerable. They take responsibility for the greater good in the longer term and preserve the planet for future generations. New leaders can learn from the COVID-19 crisis that there is a need to put the collective interest above the individual interest. To quote another Nobel Prize Winner, Nelson Mandela: “For to be free is (…) to live in a way that respects and enhances the freedom of others.”

The challenge for the leaders of today and tomorrow is to navigate the myriad of interests of the various stakeholders’ responsibly. Universities have a critical contribution to make in educating the next generation of leaders to critically reflect on the pressing issues that confront us all. Tilburg University has a starring role to play in producing the responsible leaders of tomorrow, who will purposely lead the acceleration to the new common.
